# Research trends and hotspots of Colles fracture: a bibliometric analysis from 1980 to 2023

**DOI:** 10.3389/fsurg.2025.1509556

**Published:** 2025-02-19

**Authors:** Chaoxi Zhou, Guangrong Yu, Qinglei Wang

**Affiliations:** ^1^ Department of Emergency Surgery, Beijing Geriatric Hospital, Beijing, China; ^2^ Department of Orthopedics, Beijing Geriatric Hospital, Beijing, China

**Keywords:** Colles fracture, nonoperative, osteoporosis, bibliometric, rehabilitation, external fixation, open reduction internal fixation

## Abstract

**Background:**

Colles fractures, a common type of distal radius fracture, predominantly affect older adults and are often associated with osteoporosis. Understanding the epidemiology, treatment methods, and complications of Colles fractures is crucial for improving patient outcomes.

**Objective:**

This bibliometric analysis aims to assess the trends, influential research, and collaboration patterns in Colles fracture studies from 1980 to 2023, providing insights into emerging areas of research.

**Methods:**

Literature was retrieved from the Web of Science Core Collection (WoSCC), Science Citation Index Expanded (SCI-EXPANDED) using the search term “Colles fracture”. A total of 948 relevant documents, including 901 articles and 47 reviews, were analyzed. VOSviewer, CiteSpace, and bibliometrix were utilized for visualization and data analysis, focusing on publication trends and hotspots.

**Results:**

The analysis revealed a steady increase in publications and citation counts, peaking around 2010, with a notable decline in publication output post-2010 while citations continued to rise. The USA led in both publication volume and citation impact, with significant contributions from England, Canada, Germany, and Japan. Key authors such as Cooney WP and Jupiter JB were identified as influential, while the Journal of Hand Surgery-American Volume emerged as the leading publication outlet. Keyword analysis indicated a growing emphasis on epidemiology and outcomes research, reflecting broader public health concerns.

**Conclusion:**

This bibliometric analysis highlights the evolving research on Colles fractures from 1980 to 2023. Despite a plateau in publication rates, citations continue to increase, indicating the lasting influence of earlier studies. Significant advancements have been made in treatment methods, particularly in external fixation (EF) and open reduction internal fixation (ORIF). The growing interdisciplinary focus on Colles fractures, osteoporosis, and rehabilitation underscores the need for continued research to enhance clinical outcomes and preventive measures.

## 1 Introduction

Colles fracture is a common type distal radius fractures, particularly prevalent among the elderly, and is often associated with low energy or traumatic events (1). Since it was first described by Abraham Colles in 1814 (2), research on this fracture has continuously expanded, particularly in the areas of fracture treatment methods, complication management, and patient rehabilitation. Over the years, significant advancements have been made in understanding the pathophysiology of Colles fractures, the optimal treatment strategies, and the long-term outcomes for patients. With the global trend of population aging and the rising prevalence of osteoporosis (3), the incidence of Colles' fractures has also increased (4). In particular, Colles' fractures are considered a hallmark of osteoporosis in older women, significantly impacting their quality of life and independence (5).

Computed Tomography (CT) and x-ray are important tools for diagnosing Colles fractures and evaluating treatment outcomes, each offering distinct advantages. X-ray remains the primary imaging modality due to its accessibility, speed, and effectiveness in detecting fractures, particularly displaced fractures and joint misalignments. However, it has limitations in visualizing soft tissue injuries and complex fractures (6). CT offers superior accuracy in assessing the fracture morphology, including comminuted fractures, and provides detailed information on bone displacement and alignment (7). It is especially valuable for pre-surgical planning and evaluating complex fracture patterns.

In terms of fracture treatment, there has been ongoing debate regarding the advantages and disadvantages of various techniques. The conservative treatment for Colles' fracture typically involves closed reduction followed by immobilization with a cast or splint (8), which may have less injury, especially reccommended for aged population. In contrast, surgical intervention, including both internal and external fixation, is preferred for complex, displaced fractures (9). Both internal fixation and external fixation techniques are widely applied in clinical practice, yet there remains debate about which method offers better functional recovery, lower complication rates, and improved patient outcomes (10–12).

Epidemiological studies have identified the risk of distal radius fractures (DRF) is influenced by personal factors such as age, sex, and health, as well as environmental factors like population density and climate, with poorer outcomes associated with older age, female sex, poor bone healing, and lower socioeconomic status (13). And currently, the field is witnessing a shift toward more comprehensive research that integrates both clinical and epidemiological perspectives. An increasing number of studies are focusing on the epidemiology of the fractures, risk factors, long-term prognosis, and their public health impact (14–16). Particularly in aging societies, the relationship between Colles' fractures and osteoporosis, as well as the importance of preventive measures for fractures, has garnered more attention (17). Additionally, research on functional recovery and long-term quality of life has gradually become a hot topic in this field (18, 19). These research findings provide valuable evidence for clinicians in formulating treatment plans and also offer strong support for the development of public health policies.

The motivation for this bibliometric study stems from the need to systematically analyze the growing body of research on Colles fractures and identify emerging trends in the field. While a large number of studies have been conducted, there is a lack of comprehensive analyses that integrate the various aspects of Colles fracture research, such as treatment methods, epidemiology, and long-term outcomes. Through the use of bibliometric tools like VOSviewer (20), Bibliometrix (21) and CiteSpace (22, 23), this study will identify influential authors, institutions, and journals, while also mapping the collaboration networks and citation landscapes that have shaped the field. Furthermore, the study will highlight key topics, such as the role of epidemiology and the evolving techniques in fracture management, offering valuable insights for future research directions in this area.

## 2 Materials and methods

### 2.1 Literature retrieval and data collection

**Figure 1:**
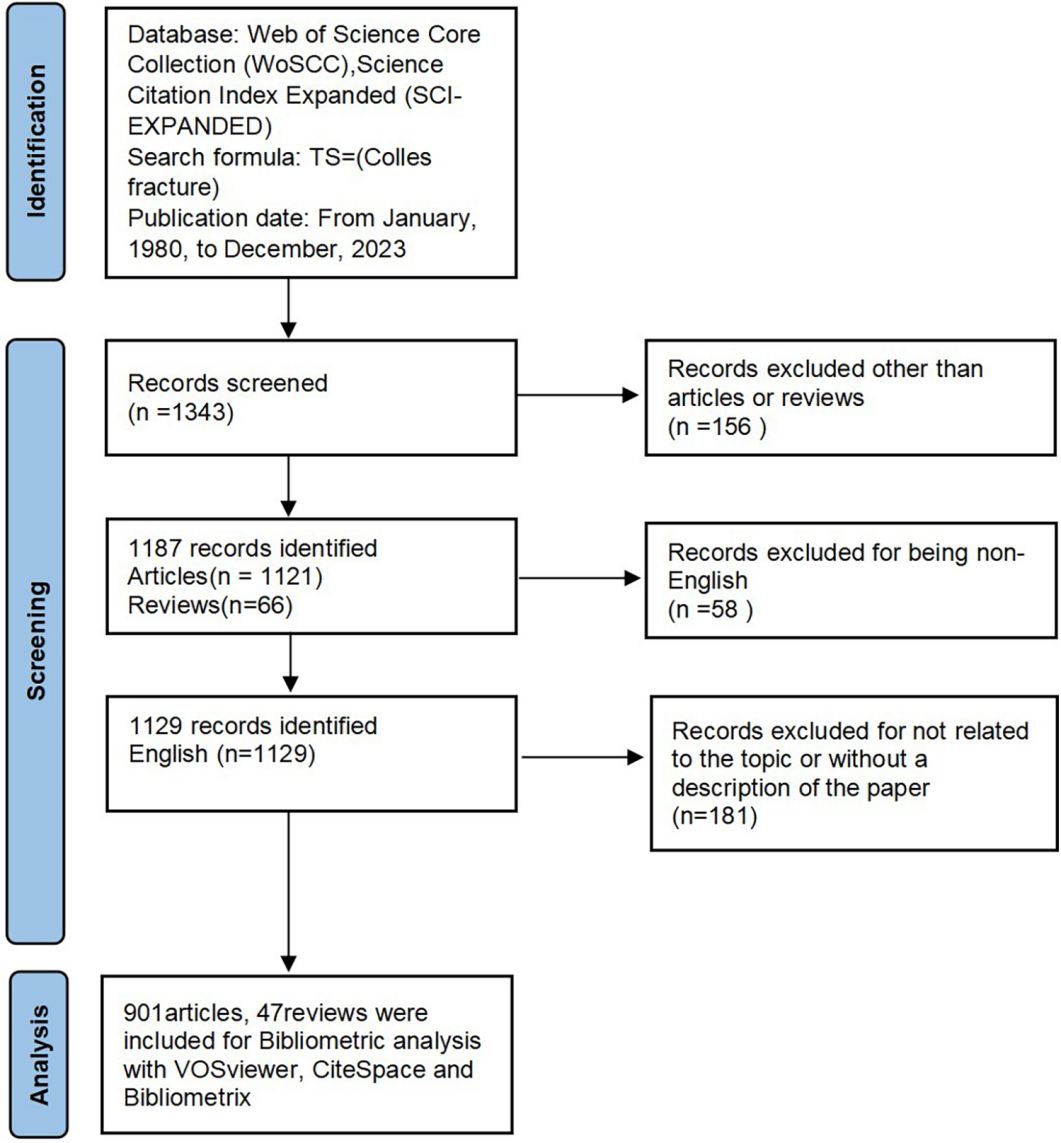
The flowchart of literature selection and bibliometric analysis.

The data for this bibliometric study on Colles fractures were retrieved from the Web of Science Core Collection (WoSCC), specifically the Science Citation Index Expanded (SCI-EXPANDED). WoSCC was chosen due to its comprehensive coverage and high accuracy in document type labeling, which is superior to other databases. The search was conducted using the following search formula: TS = (Colles fracture). The publication date was restricted from January 1, 1980, to December 31, 2323, ensuring a comprehensive range of research coverage. Only documents classified as articles or reviews and published in English with a description of research and results were considered. All search operations and data retrieval were completed on a single day, September 7, 2024, to reduce any inconsistencies arising from possible database updates. Two reviewers worked independently assess the retrieved publications to ensure the relevance to the research topic. Finally, a total of 948 records from 1980 to 2023, comprising 901 research articles and 47 reviews, all preserved in plain text format (Figure 1).

### 2.2 Data analysis

The visualization of authors and research institutions was performed using VOSviewer V1.6.20, a widely-used bibliometric tool designed for constructing and visualizing bibliometric networks. The collaboration network between countries or regions was mapped using Scimago Graphica, which allows for the intuitive representation of research collaborations. CiteSpace V6.2.R3 was employed for data cleaning and analysis, including dual-map overlay, keyword analysis, and burst detection, providing insights into emerging trends. Additionally, we utilized bibliometrix, an R package for comprehensive bibliometric analysis, to generate the Three Fields Plot and examine journal publication trends.

In these visual representations, each node represents a distinct bibliometric element, such as a country, institution, author, or keyword. The size of the node reflects the frequency or weight of the element, with larger nodes indicating higher significance. Both nodes and connecting lines are color-coded to represent different clusters or time periods. The thickness of the lines indicates the strength of the relationships or collaborations between the nodes. These tools, commonly used in scientometrics, offer a comprehensive framework for understanding the structure and dynamics of scholarly communication.

## 3 Results

### 3.1 Publication volume and growth trends

**Figure 2:**
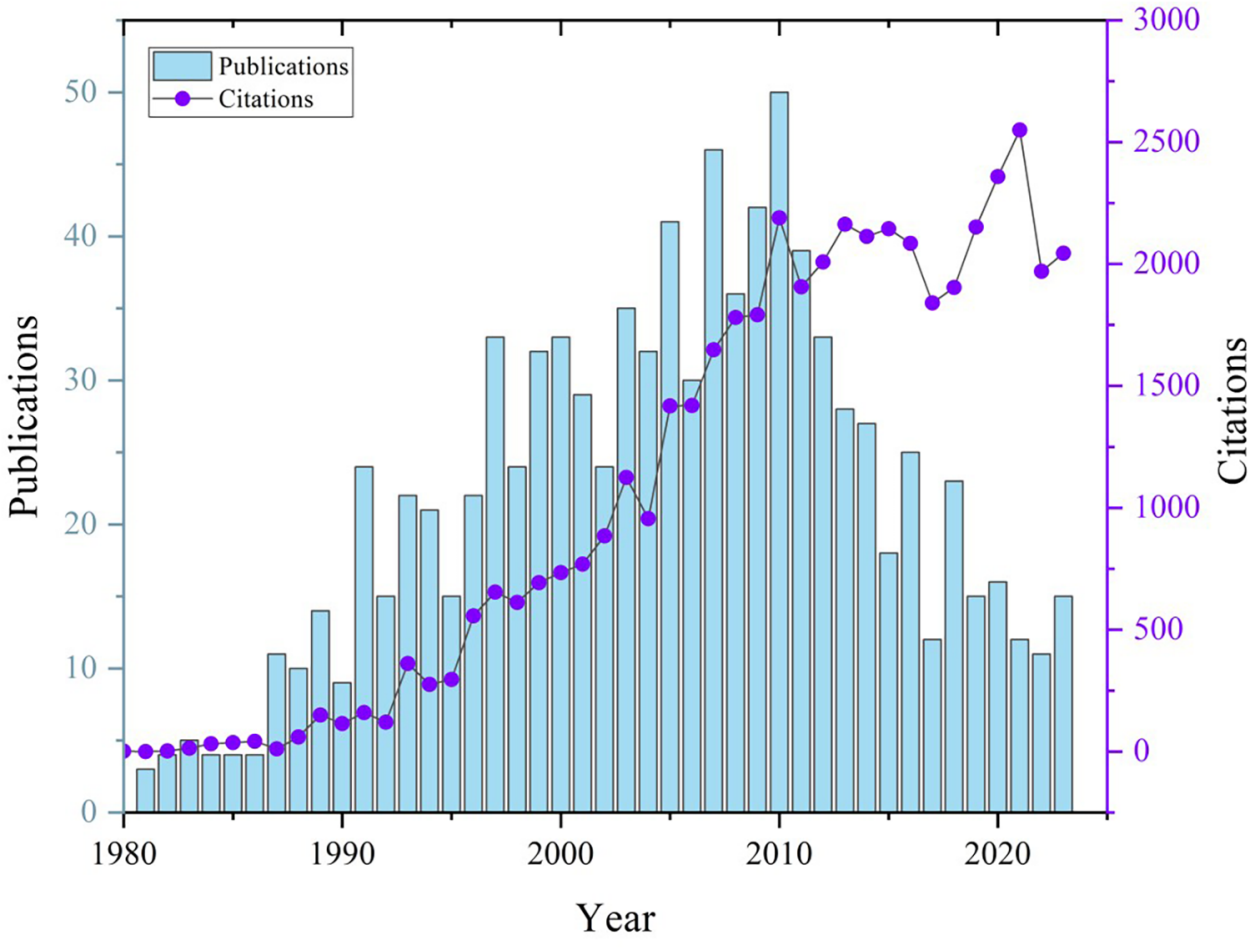
The international annual publication and citation trend of research about Colles fracture.

From 1980 to the early 2000s, both publication output and citation counts increased steadily, reflecting the growing scholarly attention to Colles’ fractures. The annual publication reached a peak of 50 papers around 2010 coincides with a significant rise in citations, indicating a period of high-impact research and major contributions to the field. Post-2010, while the number of publications declined, citation counts remained keep a upward trajectory, with a secondary peak observed around 2020 (Figure 2). This suggests that despite the reduced number of new studies, existing literature continued to be influential and widely cited.

### 3.2 Country and institutional analysis

**Figure 3:**
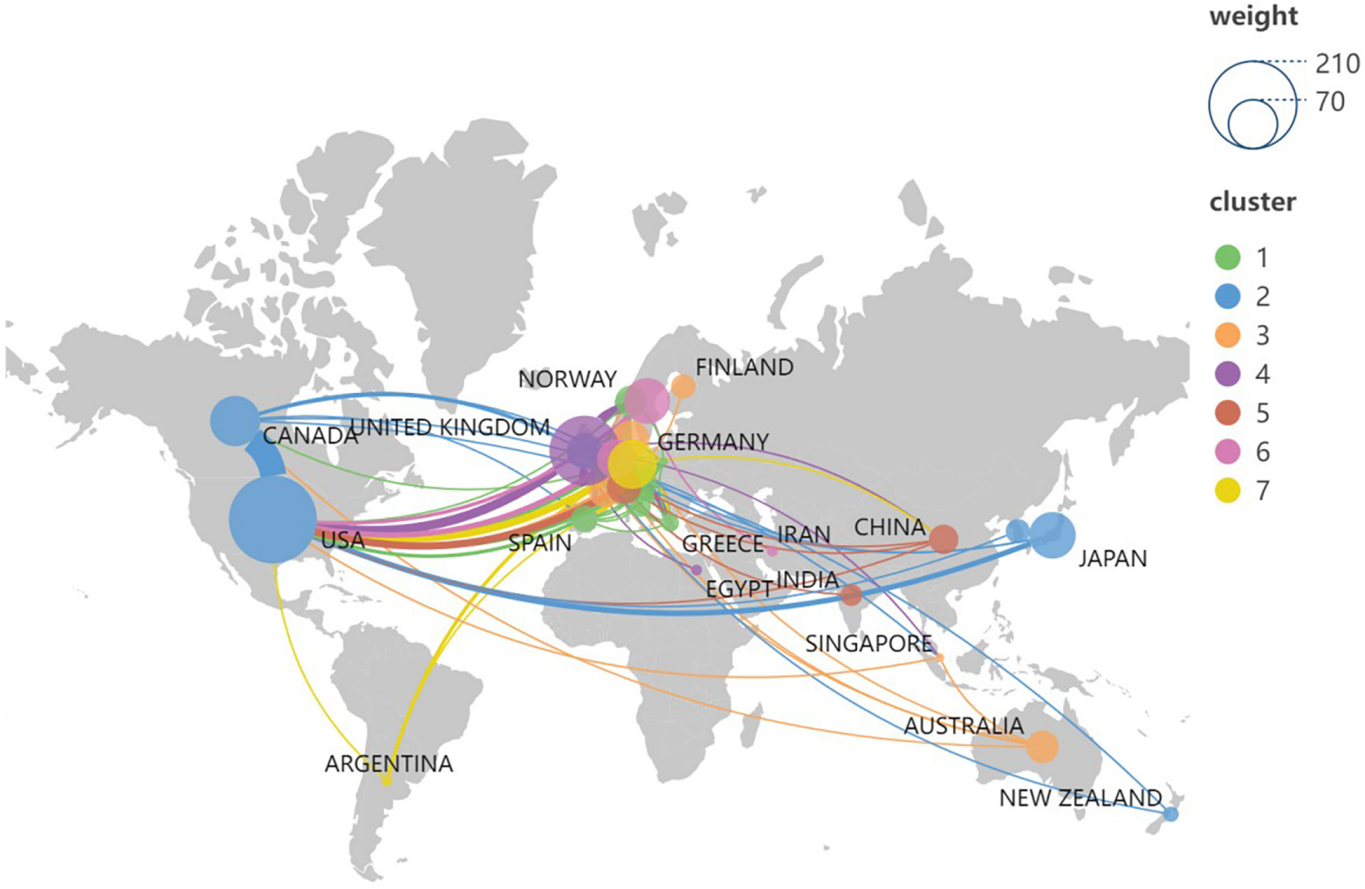
Country/region distribution and international networking of global publications in Colles fracture.

From 1980 to 2023, global research on Colles fractures was dominated by the USA, which contributed the most publications and received the highest number of citations (12,249). England, Canada, Germany, and Japan also made significant contributions, with England publishing 114 articles and receiving 5,304 citations. Although countries like Sweden and the Netherlands had fewer publications, their citation-per-article ratios were notably high (72.06 and 70.41, respectively), indicating a strong impact of their research (Table 1). Collaboration between countries was robust, particularly between North America and Europe, with the USA, UK, Canada, and Germany forming the core of international research efforts (Figure 3).

**Table 1:** The top 10 countries/regions making the most significant contributions to the field of Colles fracture.

Rank	Country/Region	Publications	Citations	Citation per article
1.00	USA	196.00	12,249.00	62.49
2.00	England	114.00	5,304.00	46.53
3.00	Canada	64.00	2,490.00	38.91
4.00	Germany	61.00	1,979.00	32.44
5.00	Japan	55.00	1,087.00	19.76
6.00	Sweden	53.00	3,819.00	72.06
7.00	Netherlands	39.00	2,746.00	70.41
8.00	Denmark	36.00	1,041.00	28.92
9.00	Scotland	32.00	1,636.00	51.13
10.00	Switzerland	30.00	1,647.00	54.90

**Figure 4:**
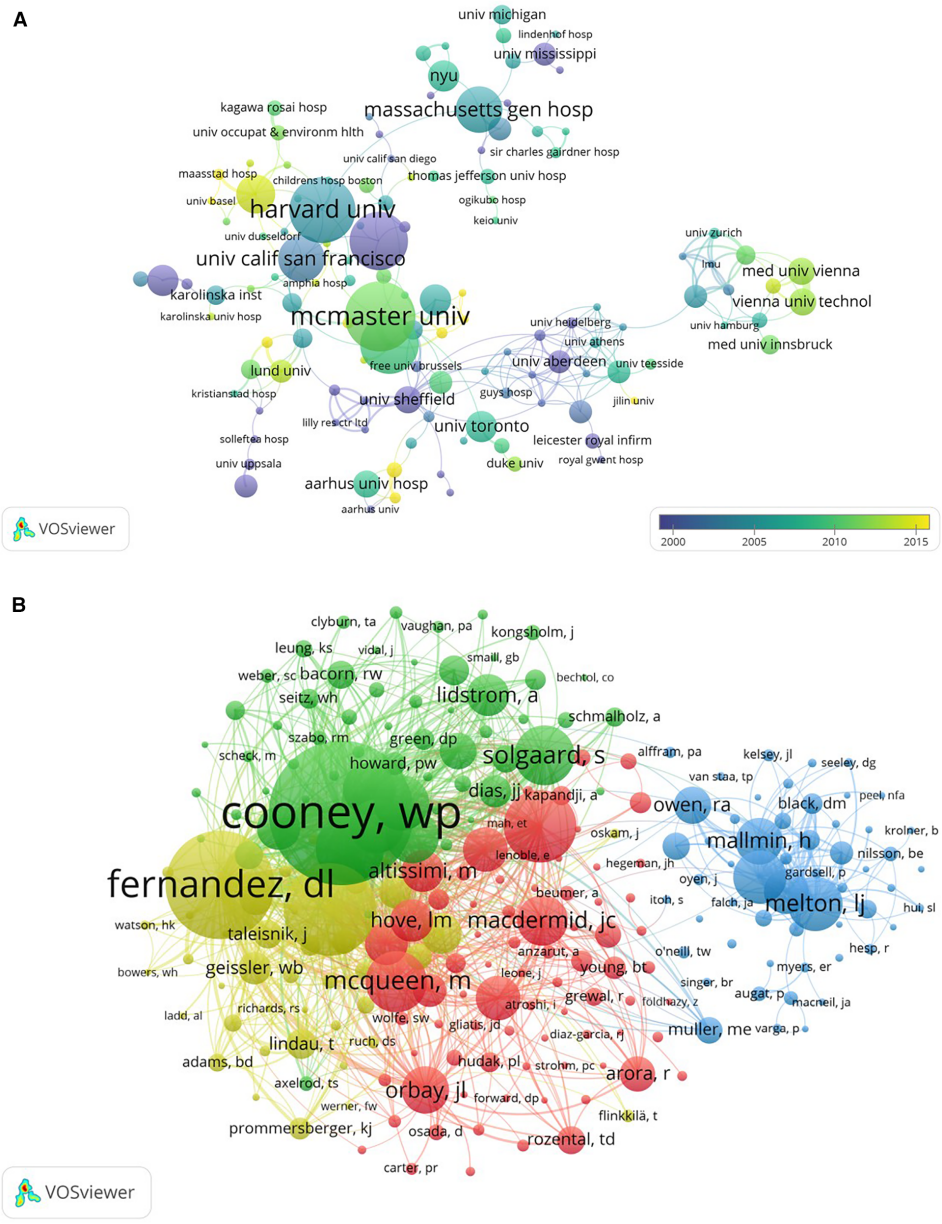
**(A)** Co-authorship analysis of institutions in Colles fracture. **(B)** Co-citation analysis of the first author.

At the institutional level, Harvard University and McMaster University played leading roles in Colles fracture research, both in terms of publication output and collaborations. Notably, University of California, San Francisco has the highest citation per article, indicating the significant impact of its publications despite having fewer overall publications (Table 2). Harvard University had extensive partnerships with other North American institutions, such as the University of California, San Francisco, and Massachusetts General Hospital. Similarly, McMaster University fostered strong ties with European and North American institutions. These connections were essential in advancing research in the field, with many partnerships strengthening over time, particularly in the late 1990s and early 2000s (Figure 4A).

**Table 2:** The leading 10 institutions in terms of publication volume.

Rank	Organization	Country/Region	Publications	Citations	Citation per article
1	McMaster University	Canada	18	902	50.11
2	Harvard University	USA	17	837	49.24
3	Mayo Clinic	USA	15	2,176	145.07
4	University of Western Ontario	Canada	15	609	40.6
5	Massachusetts General Hospital	USA	12	897	74.75
6	University of California, San Francisco	USA	12	2,409	200.75
7	University of Amsterdam	Netherlands	10	164	16.4
8	Danderyd Hospital	Sweden	8	375	46.88
9	New York University	USA	8	346	43.25
10	University of Toronto	Canada	8	278	34.75

### 3.3 Author analysis

A total of 3,250 authors have contributed to research on Colles' fractures. Based on a co-citation cluster analysis (Figure 4B), leading the field in terms of co-citations is Cooney WP (green), followed by Fernandez DL (yellow), Mcqueen M (red) and Melton LJ (blue). The co-citation map reveals strong collaborative networks among each cluster. For example, Cooney WP from the green cluster have frequent cross-citations with McQueen M (red) and Fernandez DL (yellow), showing a tight-knit academic relationship. Solgaard S (green) and Macdermid JC (red) also share a significant citation overlap, indicating a close intellectual partnership. These relationships illustrate how foundational research by key authors continues to shape the direction of ongoing studies in the field of Colles fracture.

The analysis of the top 10 most prolific authors in Colles fracture research (Table 3) reveals that Jupiter JB (USA) leads in publication output with 11 papers and a significant citation count of 855, averaging 77.73 citations per article. However, Melton LJ (USA) and John A. Kanis (England) stand out with fewer publications (9 and 8, respectively) but exceptionally high citation counts of 1,935 and 1,864, translating to 215 and 233 citations per article, respectively, indicating their profound influence on the field. Other notable contributors include McQueen MM (Scotland) and Grewal Ruby (Canada), while authors from Sweden also show steady contributions, though with lower citation averages.

**Table 3:** The top 10 most prolific authors in Colles fracture research.

Rank	Author	Country/Region	Publications	Citations	Citation per article
1	Jupiter JB	USA	11	855	77.7273
2	Hove LM	Norway	10	323	32.3
3	Field J	England	9	283	31.4444
4	Grewal Ruby	Canada	9	359	39.8889
5	Melton LJ	USA	9	1,935	215
6	Abbaszadegan H	Sweden	8	301	37.625
7	Jonsson U	Sweden	8	291	36.375
8	Kanis JA	England	8	1,864	233
9	Macdermid Joy C	Canada	8	334	41.75
10	McQueen MM	Scotland	8	645	80.625

### 3.4 Journal analysis

This analysis (Table 4) highlights the top 10 journals with the highest number of publications in the field of Colles' fractures. The Journal of Hand Surgery-American Volume ranks first with 100 publications, accumulating 4,914 citations and an average of 49.14 citations per article, with a 2023 impact factor (IF) of 2.1 (Q2). Injury-International Journal of the Care of the Injured and Acta Orthopaedica follow with 62 and 39 publications, respectively, and citation per article values of 23.02 and 32.49. Their 2023 IF are 2.2 (Q2) and 2.5 (Q1). Osteoporosis International, despite having 39 publications, stands out with a high citation per article count of 98.72 and a 2023 IF of 4.2 (Q1). These results emphasize the prominence of orthopedic and trauma journals in Colles' fracture research, with notable contributions from clinically and surgically focused journals.

**Table 4:** The top 10 journals with the highest number of publications in the field of Colles fracture.

Rank	Journal	Publications	Citations	Citation per article	IF(2023)	JCR(2023)
1	Journal of Hand Surgery-American Volume	100	4,914	49.14	2.1	Q2
2	Injury-international Journal of The Care of the Injured	62	1,427	23.02	2.2	Q2
3	Acta Orthopaedica	39	1,267	32.49	2.5	Q1
4	Journal of Hand Surgery-European Volume	39	1,177	30.18	2	Q2
5	Osteoporosis International	39	3,850	98.72	4.2	Q1
6	The Journal of Bone and Joint Surgery-British Volume	37	3,260	88.11	4.9	Q1
7	Archives of Orthopaedic and Trauma Surgery	34	998	29.35	2	Q2
8	Journal of Bone And Joint Surgery-American Volume	33	3,207	97.18	4.4	Q1
9	Journal of Hand Surgery-European Volume	28	513	18.32	2	Q2
10	Journal Of Orthopaedic Trauma	26	761	29.27	1.6	Q3

**Figure 5:**
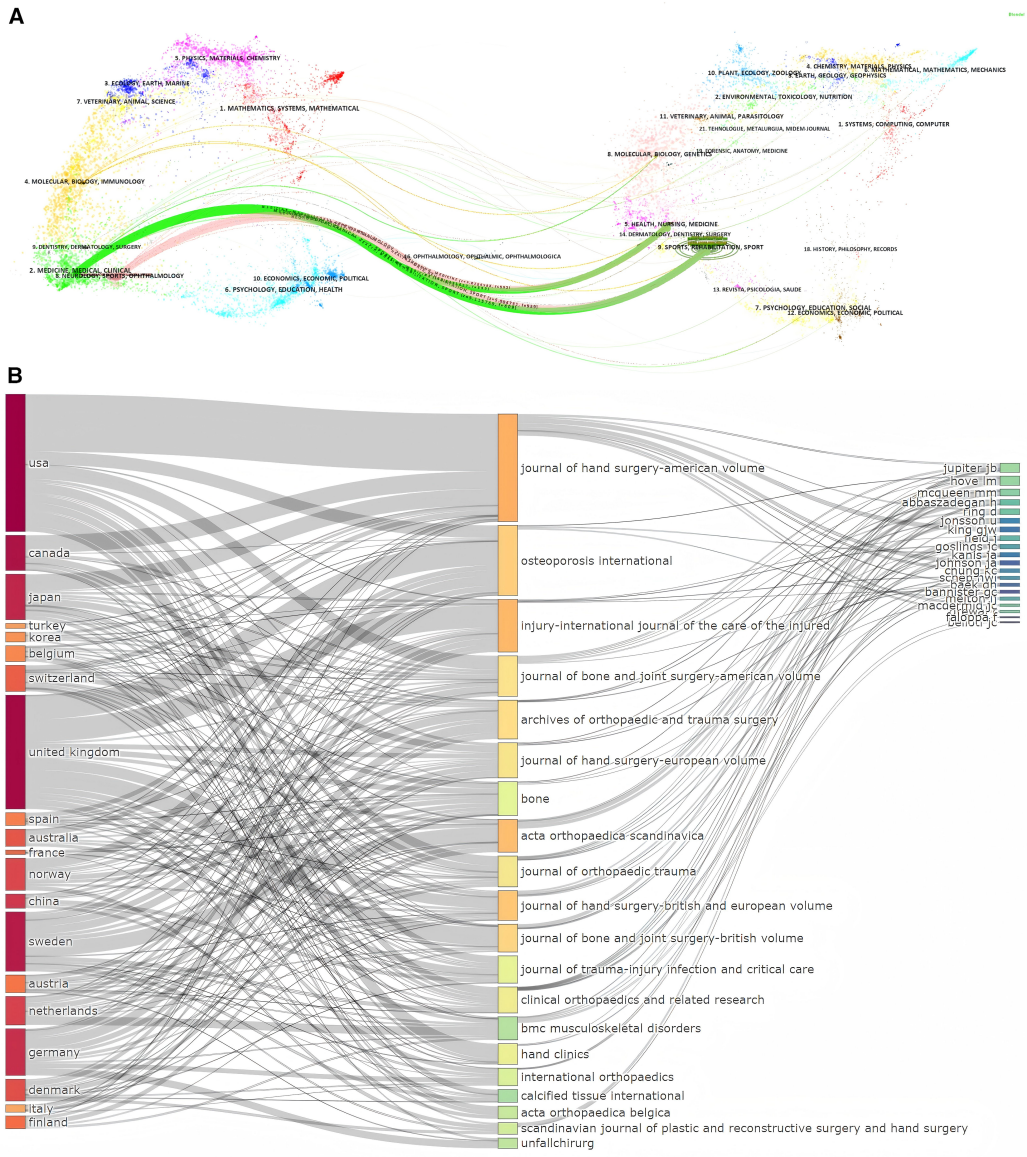
**(A)** Dual-map overlay of journals about Colles fracture. **(B)** The three fields plot.

In Figure 5A, the dual-map overlay visualizes the citation landscape of the journals included in the study. The map on the left side represents the journals cited by the articles in the study, while the map on the right depicts the journals from which the citations originate. The overlay reveals 2 primary citation paths, with the pink citation path exhibiting the highest *z*-score. This prominent path signifies that articles in the field of “Neurology, Sports, Ophthalmology” predominantly cited literature in the “Sports, Rehabilitation, Sport” domain. This suggests a strong interdisciplinary linkage where clinical research of colles fracture draws extensively from sports and rehabilitation studies, emphasizing the interconnectedness of these scientific domains in advancing medical knowledge.

The three-field plot (Figure 5B) summarizes the relationships between countries, journals, and authors. The USA, as the country with the highest number of publications, has most of its research published in The Journal of Hand Surgery-American Volume. In contrast, the UK, which ranks second in terms of publication volume, shows a more dispersed pattern across various journals. The Journal of Hand Surgery-American Volume is the most frequently targeted journal, while other key journals include Osteoporosis International and Injury-International Journal of the Care of the Injured. Prominent authors such as Jupiter JB, Hove IM and McQueen MM are identified as significant contributors, showcasing a strong international collaboration in this field.

### 3.5 Reference analysis

**Figure 6:**
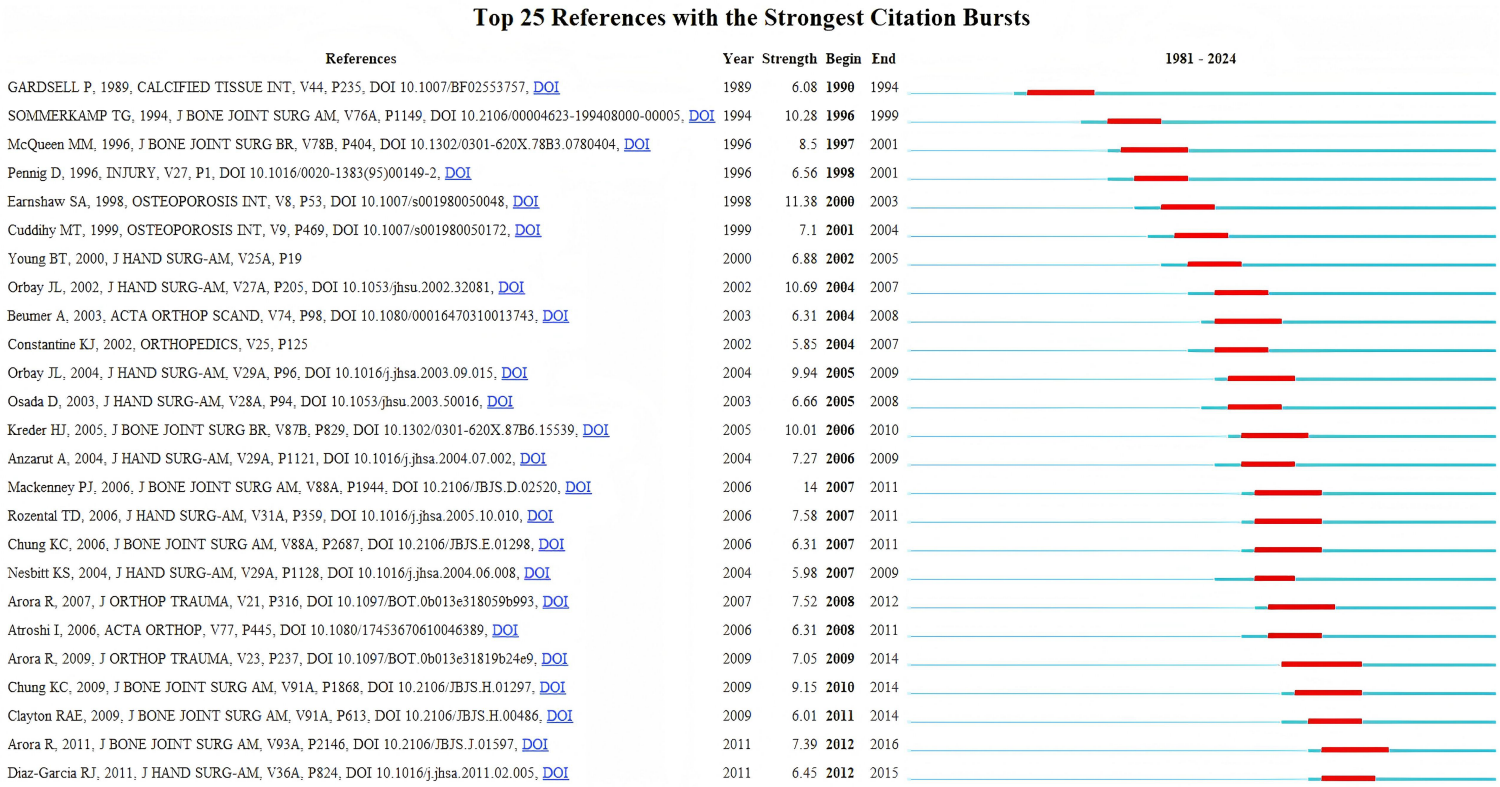
Top 25 references with the strongest citation bursts.

The Figure 6 shows the top 25 references with the strongest citation bursts in Colles' fracture research in this work. The citation burst strength indicates the intensity of attention these references received over time. The highest burst, 11.38, was observed for a study by Earnshaw SA published in Osteoporosis International, peaking between 2000 and 2003. The study found that osteoporosis and high bone turnover are common in postmenopausal patients with Colles' fractures, with younger patients (aged 65 or less) showing lower-than-expected BMD at the hip (24). Another notable burst was from Orbay JL in Journal Of Hand Surgery-american Volume, with a strength of 10.69 between 2004 and 2007. His work reported that using a volar approach with a fixed-angle internal fixation device for treating dorsally displaced, unstable distal radial fractures resulted in excellent radiographic and functional outcomes (25). Several other studies, such as Kreder HJ (burst strength 10.01) and Sommerkamp TG (burst strength 10.28), also showed significant bursts. Kreder HJ et al. reported that indirect reduction and percutaneous fixation resulted in faster functional recovery and superior outcomes compared to open reduction and internal fixation for displaced intra-articular distal radius fractures (26). Similarly, Sommerkamp TG et al. found that static external fixation produced better clinical outcomes and fewer complications than dynamic external fixation in the management of unstable distal radius fractures (27).

### 3.6 Keywords analysis

**Figure 7:**
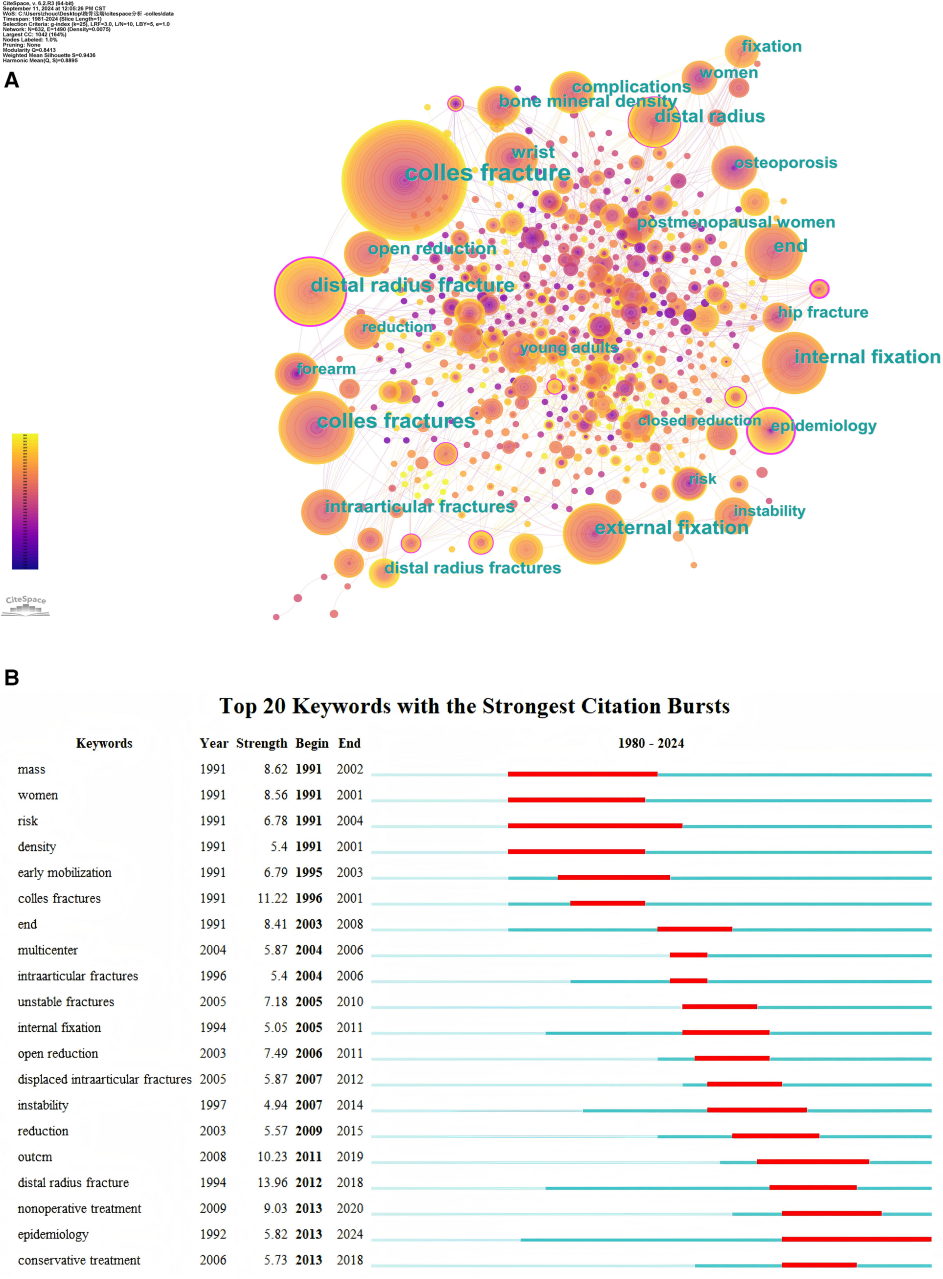
**(A)** Co-occurrence map of keywords. **(B)** Top 20 keywords with the strongest citation bursts.

In this study, a keyword co-occurrence map was generated using CiteSpace to analyze research trends related to Colles fractures (Figure 7A). The network map visualizes the relationships between keywords extracted from the relevant literature, with different colors representing keywords emerging from different time periods. The presence of purple halos around certain nodes indicates high centrality values (greater than 0.1), suggesting that these terms serve as pivotal connecting points in the research network. Key terms such as “Colles fracture (centrality value 0.23)”, “distal radius fracture (centrality value 0.16)”, “distal radius (centrality value 0.14)”, “epidemiology (centrality value 0.13)”, and “external fixation (centrality value 0.11)” are central, indicating their significant frequency and relevance in the literature. Other notable nodes include keywords related to osteoporosis, internal fixation, and complications, reflecting ongoing clinical interest in the treatment and complications associated with Colles fractures.

The analysis of the top 20 keywords with the strongest citation bursts related to Colles fractures is presented in Figure 7B. Citation bursts indicate periods when specific hotspot gained considerable attention in academic literature. The highest burst strength was observed for the keyword “distal radius fracture” (strength = 13.95), which began in 2012 and ended in 2018. Another significant citation burst was associated with the keyword “colles fractures” (strength = 11.21), spanning from 1996 to 2001. Other notable keywords include “outcm” (outcomes), which had a strength of 10.22 from 2011 to 2019. Particularly noteworthy is the citation burst for “epidemiology” (strength = 5.82), which began in 2013 and is still ongoing, extending through 2024. The sustained nature of this burst suggests that epidemiology may continue to be a focal point for future research in Colles fractures.

## 4 Discussion

### 4.1 General information

This bibliometric work revealed that the evolution of colles fracture is dynamic. The steady increase in both publication output and citation counts, particularly during the 1990s and early 2000s, indicates a growing scholarly interest in Colles fractures. Although publication numbers have plateaued since 2010, the upward trajectory of citation counts, with a notable peak around 2020, suggests that existing studies continue to be highly influential. This trend may reflect the consolidation of critical knowledge and treatment advancements that remain relevant in ongoing clinical practice.

The analysis of countries and institutions involved in Colles fracture research highlights the USA's dominant position, contributing the highest number of publications and receiving the most citations. England, Canada, Germany, and Japan also made significant contributions, with strong collaborative networks between North America and Europe. High citation-per-article ratios from countries like Sweden and the Netherlands suggest the impactful nature of their research despite fewer publications. The prominence of leading institutions, such as Harvard University and McMaster University, further underscores the importance of international collaboration, particularly between North America and Europe, in advancing research in this field.

The analysis of key researchers highlights the foundational contributions of Cooney WP, McQueen MM, and Fernandez DL, whose collaborative efforts have significantly shaped the field of distal radius fractures. Cooney WP's research primarily focuses on understanding the biomechanical effects of these fractures, optimizing both internal and external fixation techniques, and investigating the implications of fracture angulation on joint kinematics and postoperative rehabilitation (28–30). Similarly, McQueen MM et al. has advanced the understanding of distal radial fractures through her work on the efficacy of external fixation, predictors of instability, and factors influencing functional outcomes, emphasizing the role of non-bridging fixation and bone quality in recovery (31–34). Fernandez DL has further enriched the field by developing classification systems and refining treatment approaches, particularly for complex fracture patterns involving the distal radioulnar joint and volar lunate facet, thus improving functional outcomes and reducing post-traumatic complications (35–37). Together, their seminal research continues to influence current studies and clinical practices.

Journal analysis indicates that the Journal of Hand Surgery-American Volume is the most significant outlet for Colles fracture research, followed by Injury-International Journal of the Care of the Injured and Acta Orthopaedica. Notably, Osteoporosis International stands out with a high citation-per-article count, highlighting the relevance of bone health and osteoporosis in Colles fractures, particularly in postmenopausal populations. The dual-map overlay further emphasizes the interdisciplinary nature of Colles fracture research, with substantial links to sports, rehabilitation, and related clinical domains.

### 4.2 Hotspots and frontiers

Epidemiological studies have demonstrated a growing incidence of Colles fractures (4), particularly among elderly populations. This trend is primarily attributed to the aging demographic and the rising prevalence of osteoporosis (3). Understanding the epidemiology of these fractures is critical for public health planning, especially in the context of fracture prevention and osteoporosis management. Korpelainen, R et al. (38) found that while impact exercise did not prevent bone mineral density (BMD) loss, it reduced bone mineral content (BMC) loss at the trochanter and lowered the risk of fall-related fractures such as Colles fracture in elderly women with low bone mass. In a population-based case-control study by Mallmin, H., heredity, nulliparity, and estrogen deficiency were identified as significant risk factors for distal forearm fractures in women, while postmenopausal estrogen therapy had a protective effect, and lifestyle factors showed no significant differences between patients and controls (39).

The debate surrounding the use of external fixation (EF) vs. open reduction internal fixation (ORIF) for colles fractures remains a focal point, with each technique presenting distinct advantages and limitations. Both methods aim to restore function and ensure stable fixation, but their application and outcomes vary depending on factors such as fracture severity and patient characteristics. A meta-analysis by Margaliot et al. (40) found no significant differences between EF and ORIF regarding wrist motion, grip strength, and pain. However, while EF is associated with higher rates of infection and hardware failure, ORIF presents complications like tendon injuries and the need for hardware removal. Kateros et al. (41) compared EF with internal fixation using a dorsal “pi” plate and found that while radiographic outcomes were better in ORIF patients, clinical outcomes were comparable between the groups. This suggests that, although ORIF may offer radiographic superiority, functional results do not significantly differ from EF. Some studies also showed promising results for volar locking plates (VLP) in ORIF procedures. Westphal et al. (42) compared the outcomes of open reduction and internal fixation (ORIF) with external fixation for distal radius fractures over a one-year follow-up, finding that palmar plate fixation yielded slightly better radiological and functional results, although most differences between groups were not statistically significant. Wei et al. (11) noted better recovery of forearm supination and restoration of volar tilt in ORIF-treated patients, though EF demonstrated superior grip strength and wrist flexion. This points to ORIF being favorable for anatomical restoration, while EF may better preserve certain functional aspects.

### 4.3 Future trends

Recent citation burst analysis based on keywords indicates that nonoperative and conservative treatments have emerged as significant research trends in the management of Colles' fractures (Figure 7). In response to these trends, we further examined recent studies focused on conservative treatment options for Colles' fractures. Caruso et al. (8) conducted a randomized trial comparing the efficacy of long (above-elbow) vs. short (below-elbow) casts in maintaining fracture reduction and improving clinical outcomes. Their study found no significant differences between the two groups in terms of radiological parameters or clinical scores, concluding that short casts are as effective as long casts for post-reduction immobilization in Colles' fractures. In a separate investigation, Saving et al. (43) compared nonoperative treatment to volar locking plate fixation in elderly patients with dorsally displaced distal radius fractures. Their results showed that volar locking plates yielded improved functional outcomes, including enhanced grip strength and wrist function, although complication rates were similar between both groups. Furthermore, a network meta-analysis by Woolnough et al. (44) assessed various surgical and nonoperative treatment options for distal radius fractures. The analysis indicated that volar plating resulted in better functional outcomes and fewer complications compared to nonoperative treatment, particularly for intraarticular fractures. However, no significant differences in functional outcomes were observed between nonoperative treatments and different surgical methods, suggesting that volar plating may be preferred for certain patient populations, especially those with intraarticular fractures or individuals aged over 60 years of age.

In general, recent citation bursts in the field of epidemiology reveal a significant shift in research on Colles' fractures, transitioning from an emphasis on surgical approaches to conservative treatments, and now moving toward a focus on prevention strategies. While current literature on robot-assisted surgery for Colles' fractures remains sparse, the increasing integration of robotic technology into orthopaedic surgical practice (45, 46) suggests that this may emerge as a prominent research area in the near future.

### 4.4 Limitations

This bibliometric study has several limitations. First, the data were sourced exclusively from the Web of Science Core Collection (WoSCC), which, while comprehensive, may not include relevant research indexed in other databases, such as Scopus or PubMed. Although it is acknowledged that relying on a single database may result in the omission of potentially relevant studies, the limitations of our research team's resources necessitated the use of WoSCC alone for this study. Second, the analysis was restricted to articles and reviews published in English, potentially excluding significant contributions from non-English publications. This language bias could limit the generalizability of our findings, especially in regions where English is not the primary language of scientific communication. Additionally, the study focuses solely on Colles fractures, excluding other key subtypes of distal radius fractures, such as Smith and Barton fractures, which limits the comprehensiveness of the analysis. Finally, the study period ends in 2023, and research trends may have shifted since, limiting the applicability of our conclusions to current and future developments in distal radius fracture research.

## 5 Conclusion

This bibliometric analysis highlights the evolving research landscape of Colles fractures from 1980 to 2023. While publication rates have plateaued in recent years, citation counts continue to rise, underscoring the sustained influence of earlier studies. The USA and leading institutions such as Harvard University have played central roles, while countries like Sweden and the Netherlands made high-impact contributions. Key researchers, including Cooney WP and McQueen MM, have significantly influenced the field, particularly in treatment approaches like external fixation (EF) and open reduction internal fixation (ORIF). The interdisciplinary connections between Colles fractures, osteoporosis, and rehabilitation emphasize the need for ongoing research to improve clinical outcomes and preventive strategies.

## Data Availability

The original contributions presented in the study are included in the article/Supplementary Material, further inquiries can be directed to the corresponding author.
